# Homogeneous Intrinsic Neuronal Excitability Induces Overfitting to Sensory Noise: A Robot Model of Neurodevelopmental Disorder

**DOI:** 10.3389/fpsyt.2020.00762

**Published:** 2020-08-12

**Authors:** Hayato Idei, Shingo Murata, Yuichi Yamashita, Tetsuya Ogata

**Affiliations:** ^1^ Department of Intermedia Studies, Waseda University, Tokyo, Japan; ^2^ Principles of Informatics Research Division, National Institute of Informatics, Tokyo, Japan; ^3^ Department of Information Medicine, National Center of Neurology and Psychiatry, Tokyo, Japan; ^4^ Department of Intermedia Art and Science, Waseda University, Tokyo, Japan

**Keywords:** neurodevelopmental disorder, computational psychiatry, aberrant precision, E/I imbalance, embodiment, recurrent neural network, learning, adaptation

## Abstract

Neurodevelopmental disorders, including autism spectrum disorder, have been intensively investigated at the neural, cognitive, and behavioral levels, but the accumulated knowledge remains fragmented. In particular, developmental learning aspects of symptoms and interactions with the physical environment remain largely unexplored in computational modeling studies, although a leading computational theory has posited associations between psychiatric symptoms and an unusual estimation of information uncertainty (precision), which is an essential aspect of the real world and is estimated through learning processes. Here, we propose a mechanistic explanation that unifies the disparate observations *via* a hierarchical predictive coding and developmental learning framework, which is demonstrated in experiments using a neural network-controlled robot. The results show that, through the developmental learning process, homogeneous intrinsic neuronal excitability at the neural level induced *via* self-organization changes at the information processing level, such as hyper sensory precision and overfitting to sensory noise. These changes led to multifaceted alterations at the behavioral level, such as inflexibility, reduced generalization, and motor clumsiness. In addition, these behavioral alterations were accompanied by fluctuating neural activity and excessive development of synaptic connections. These findings might bridge various levels of understandings in autism spectrum and other neurodevelopmental disorders and provide insights into the disease processes underlying observed behaviors and brain activities in individual patients. This study shows the potential of neurorobotics frameworks for modeling how psychiatric disorders arise from dynamic interactions among the brain, body, and uncertain environments.

## Introduction

Brain function and its disruption involve complex interactions between multiple functional levels, from genes to molecules, neurons, neural networks, cognition, and behavior. For decades, many observations and theories of psychiatric disorders have been reported in these domains, but the clinical contributions are limited due to a lack of explanations bridging neurobiology and symptoms (1). One of the challenges in closing the gap between neurobiology and symptoms is that some clinical symptoms partly overlap among different psychiatric disorders. For example, autism spectrum disorder (ASD) is characterized by multifaceted symptoms (e.g., deficits in social communication, behavioral inflexibility, unusual sensory sensitivity, motor clumsiness, and reduced generalization) (2–4). However, behavioral inflexibility, a typical symptom of ASD, can also be observed in schizophrenia and obsessive compulsive disorder (5, 6). In addition, psychiatric disorders partly share putative neural-level and computational-level characteristics (7, 8). These facts may mean that the mappings between different levels are not one-to-one (i.e., different factors can cause seemingly similar symptoms, and a certain factor can cause different symptoms) (1).

Computational psychiatry is an emerging field poised to narrow the explanatory gap between various levels (9–11). By computationally reproducing “abnormal” perceptions and behaviors *via* models of brain functioning, the aim is to develop frameworks that enable inference of the mechanisms underlying observed behaviors and brain activities in individual patients and that provide a basis for predicting treatment effects and detecting subgroups. Previous computational modeling studies have mostly focused on simulating psychiatric symptoms on computers (12–14), but have not thoroughly considered interactions with physical environments and the concept of embodiment (15). Because human behavior involves dynamic interactions among the brain, body, and environment, a modeling approach using a physical robot might have potential for elucidating the mechanisms underlying psychiatric symptoms (16–18). In particular, use of a robot controlled by a neural network (neurorobotics) allows consideration of both developmental learning processes and real-time adaptation to changing physical environments. The aim of this study was to provide a novel explanation spanning neural, cognitive, and behavioral characteristics of psychiatric disorder by using a neurorobotics framework. This study mainly focuses on neurodevelopmental disorders, particularly ASD, because it may be a suitable target for the developmental learning framework and considerable knowledge of ASD has been accumulated at the neural, cognitive, and behavior levels.

At the cognitive level, computational theories of neurodevelopmental disorders have been well investigated in a predictive coding framework (19, 20) (a normative computational model of cognitive functions). Predictive coding explains how the brain infers causes of sensory inputs and acquires knowledge and skills as a process of updating an internal hierarchical model of the world by minimizing differences between real and predicted sensory inputs. Within this framework, precision of sensory information and precision of prior belief (prediction) determine how much prediction errors cause the brain to update its prediction. It has been proposed that various psychiatric symptoms are associated with aberrant precision of sensory information or prior belief (8, 10, 21–25). For example, excessively high precision-weighting of prediction error, which is caused by overly high sensory precision (under-estimated sensory uncertainty) or low belief precision, results in perceptions dominated by highly detailed aspects of sensory information with difficulties in extracting abstract meaning. The consequent sensory overload, or overfitting to sensory noise, can uniformly explain ASD symptoms, including deficits in social interaction and hypersensitivity (21–23).

At the neural level, multiple lines of evidence from genetic, postmortem, and animal model studies have suggested strong links between neurodevelopmental disorders including ASD and altered functioning of excitatory and inhibitory synapses or ion channels (7, 26–28). Synaptic and channel dysfunctions may critically affect regulation of ion balance in a neuron, which sets its excitability, leading to altered network excitability. In particular, it has been proposed that an increased or decreased excitatory/inhibitory (E/I) ratio and the consequent network hyper- or hypo-excitability may be associated with neurodevelopmental disorders (29–31). However, recent studies suggest that reported relationship between the direction of E/I imbalance and ASD is inconsistent (7, 32), and both excitation and inhibition can be altered, such that altered neural excitability may not be adequately reflected by the E/I ratio alone (33). Thus, neurobiological studies may posit altered neural excitability in neurodevelopmental disorders, but specific factors and mechanisms associated with symptoms remain unclear. Although a previous neurorobotics study has investigated the relationship between unusual sensory precision (uncertainty) and behavioral inflexibility (17), the comprehensive relationship among altered neural excitability, aberrant sensory precision, and psychiatric symptoms remain unclear.

To clarify the relationships among them during the developmental learning process, we hypothesize that symptoms of neurodevelopmental disorders can be explained as a disruption, caused by altered neural excitability, of efficient temporal coding. In particular, this study focuses on heterogeneity (neuron-to-neuron variability) of neuronal excitability because it has been suggested that appropriate heterogeneous neuronal excitability (firing threshold) is important for efficient coding (34–36). Alterations in the heterogeneity of intrinsic excitability might thus disrupt efficient temporal coding. This study investigated the effects of heterogeneity of intrinsic neuronal excitability on learning and subsequent real-time adaptation in dynamic uncertain environments. To test behavioral flexibility and ability in generalizing learned skills as well as motor control we used a ball-pass interaction task examined in a previous study (17). In the experiment, robots controlled by neural network models with distinct heterogeneity of intrinsic neuronal excitability were first trained to generate multiple visuomotor patterns. Then, the trained robots interacted in real time with an experimenter. In the experiment, the robots were required to generalize learned behaviors and flexibly switch their behavior by recognizing unpredictable environmental changes. The experimental results show that the robots controlled by homogeneous networks exhibited behavioral inflexibility, low generalization ability, and motor clumsiness, despite being able to mentally reproduce learned visuomotor patterns. In addition, analyses of trained neural networks suggest these behavioral alterations might result from overfitting to sensory noise (i.e., underestimated sensory uncertainty) and alterations in hierarchical neural representation of the visuomotor patterns. These results may indicate abductive reasoning consistent with the accumulated knowledge about ASD or other neurodevelopmental disorders at the neural, cognitive, and behavioral levels.

## Results

### Robot Experiment

#### Neurorobotics Framework

**Figure 1:**
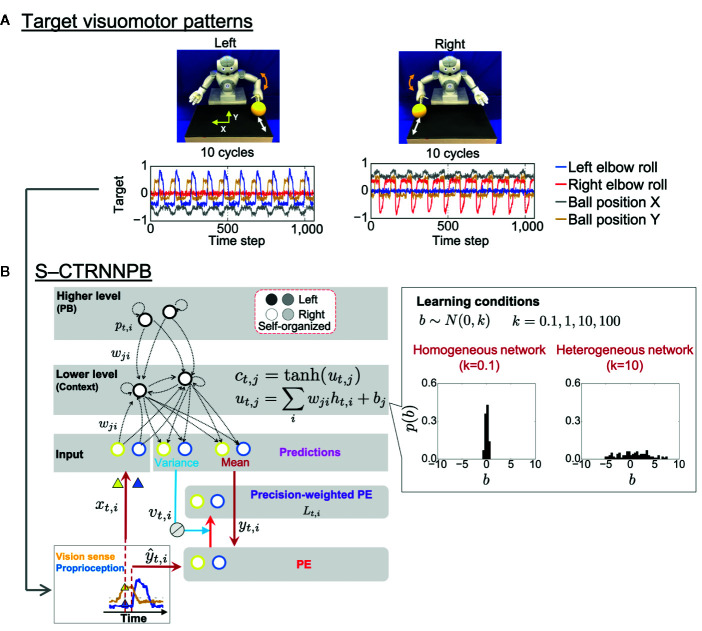
Task design and computational framework. **(A)** Two ball-pass behaviors of the robot. Time-series represent the target visuomotor sequences the stochastic-continuous time RNN with parametric bias (S-CTRNNPB) learned to reproduce. Of 10 sensory dimensions, 4 are shown. **(B)** The S-CTRNNPB utilized in this study has five groups of neurons: input, context, mean, variance, and PB. Input neurons receive current sensory inputs *x_t_*. Based on the inputs, context activity *c_t_*(lower-level sensory association), and PB activity *p_t_*(higher-level intention), the S-CTRNNPB generates predictions about the mean *y_t_*and variance *v_t_*(uncertainty) of future inputs. Parameters, such as synaptic weights *w* and the internal state of PB neurons, are optimized by minimizing precision-weighted (inverse variance-weighted) prediction error *L_t_*calculated using predictions about sensory states *y_t_*, their variance *v_t_*, and actual target sensory states *ŷ_t_*. For simplicity, the time-constant term in the equation of internal states *u_t_*of context neurons is omitted from the figure, and *h_t_*represents synaptic inputs. Bias *b* characterizes the intrinsic excitability of each neuron. The neuron-to-neuron variability of the bias *b* of context neurons was manipulated to set the heterogeneity of intrinsic neuronal excitability, and the biases were not updated during learning. The shown distribution of bias in each network condition is the average from eight networks. PE, prediction error.

In the experiment, a humanoid robot controlled by a recurrent neural network (RNN) model performed a ball interaction with a human experimenter. The neural network model used in the experiment is a version of a hierarchical predictive coding model called a stochastic-continuous time RNN with parametric bias (S-CTRNNPB) (37, 38). During task execution, the S-CTRNNPB receives sensory inputs *x_t_*comprising eight-dimensional proprioception of joint angles and two-dimensional vision of the ball position. From the inputs and context information stored in the context neurons *c_t_*and parametric bias (PB) neurons *p_t_*, the S-CTRNNPB generates predictions about the mean *y_t_*and variance *v_t_*(uncertainty) of future inputs. The robot acts according to the mean proprioceptive prediction. The robot controlled by the S-CTRNNPB first learned two types of visuomotor patterns, temporal sequences for which were prepared in advance (**Figure 1A**). In the “right” and “left” behaviors, the robot was required to wait for the ball from the experimenter and then return it, following a rule associated with ball positions. The S-CTRNNPB learned to reproduce the target visuomotor patterns by repeatedly updating the synaptic weights *w* and internal states of PB neurons to minimize the precision-weighted prediction error *L_t_*accumulated through the time length of the target sequences (**Figure 1B**). Through the learning process, context activity comes to represent short-term visuomotor patterns, and the associations between specific patterns of PB activity and corresponding target sequences are self-organized. In this sense, PB activity corresponds to a higher-level neural representation of abstract characteristic of ongoing behavior, while context activity corresponds to a lower-level neural representation of sensory association. Thus, PB activity is regarded here as the higher-level “intention” of the robot. We prepared six sequences (3 training datasets and 3 test datasets) for each behavioral pattern. In addition, random noise with a Gaussian distribution *N*(0,0.002) was added to the prepared sequences at each time step for all dimensions to provide a baseline of sensory variance for the S-CTRNNPB to estimate in learning.

**Figure 2:**
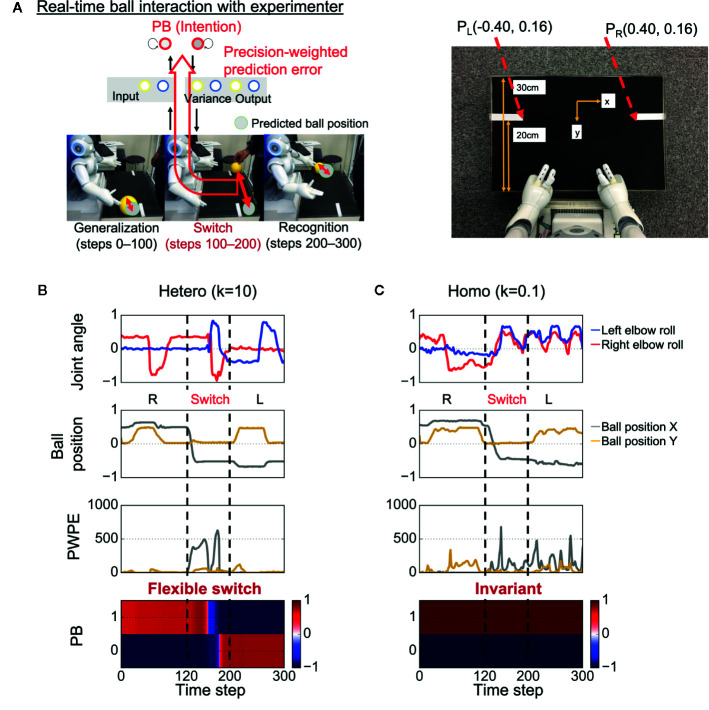
Test of behavioral generalization and flexibility in real-time ball interaction. **(A)** Experimental design of the real-time interaction. The experiment consists of three phases: generalization, switch, and recognition phases. Parametric bias (PB) activity was optimized in real time through a trial while other parameters were fixed. The robot was required to not only generate learned behaviors but also flexibly update intention (PB activity) in response to visual precision-weighted prediction errors. PL (PR), the coordinate of the inner end of the left (right) white line as captured by the robot’s camera. **(B)** An example of a successful interaction in a heterogeneous condition. The robot was able to successfully return the ball to the partner during time steps 0–100 and flexibly switch its intention toward minimizing sensory precision-weighted prediction error generated by an environmental change. **(C)** An example of behavioral alterations in a homogeneous condition. The robot failed to return the ball to the partner during time steps 0–100. In addition, the robot could not switch its intention in response to the environmental change, although the robot experienced huge precision-weighted prediction errors. The invariant intention led to abnormal periodic action during time steps 200–300. “Joint angle” indicates the mean prediction of joint angle (2 of 8 joint angles are shown). “Ball position” indicates vision inputs of ball position. “PWPE” indicates precision-weighted prediction error for vision sense after updating PB activity. “PB” indicates the activity of PB neurons. “Hetero” and “homo” indicate heterogeneous and homogeneous cases, respectively. R, right; L, left.

After learning, the trained robot interacted in real time with an experimenter for the test phase, with the robot updating its PB activity (intention) in real time, aiming to minimize visual precision weighted prediction errors (synaptic weights were fixed) (**Figure 2A**). In the test phase, the sensory experiences (e.g., the timing and speed of the ball) were not exactly the same as the training data because of the intrinsic uncertainty of the environment. In addition, unexpected changes in the ball position were induced by the experimenter. The goal of the robot was to generalize learned behaviors and flexibly recognize environmental changes by switching PB activity (intention).

#### Simulation of Altered Heterogeneity of Intrinsic Neuronal Excitability

Altered heterogeneity of intrinsic neuronal excitability was simulated by manipulating neuron-to-neuron variability in the values of bias, *b*, the parameter that determines the intrinsic excitability of each neuron (**Figure 1B**). The biases of context neurons were initialized with random values following a Gaussian distribution *N*(0*, k*) (*k* ∈{0.1, 1,10, 100, 1000}) and were not updated during learning. Here, the parameter *k* indicates the heterogeneity of intrinsic neuronal excitability. We manipulated the variance *k* from one hundredth to 100-fold of *k* = 10, a value close to the variance in the firing threshold found in cortical neurons (39, 40). We obtained results from eight trained networks with different initial synaptic weights for each setting of *k*. With *k* = 1000, the networks could not successfully learn target sequences, so the acquired data were omitted from the analysis.

### Real-Time Interaction

**Figure 2B** shows an example of successful real-time interaction in a heterogeneous condition (*k* = 10) (see also **Supplementary Video S1**). The robot first successfully performed the correct interaction with the experimenter during time steps 0–100. The fact that the robot could perform the learned task in the real environment, where the visuomotor experiences depend on the physical interactions between the robot and the object, indicates the robot could generalize the learned visuomotor sequence. Then, during time steps 100–200, the situation (ball position) was switched by the experimenter (the switch occurred at time step 120). Due to the situation switching, a discrepancy between the situation and the robot’s intention caused a strong precision-weighted prediction error signal. However, the robot could flexibly switch its intention in the direction of minimizing the generated precision-weighted prediction error, resulting in the successful interaction in the subsequent left interaction during time steps 200–300. This suggests that the robot could flexibly recognize the environmental change.

In contrast, in a homogeneous condition (*k* = 0.1), alterations in the performance were observed (**Figure 2C** and **Supplementary Video S2**). During time steps 0–100, the robot attempted to generate learned behavior but failed to return the ball to the experimenter. This suggests low generalization ability of the robot. In addition, the robot’s intention was invariant through the trial, although the robot experienced huge precision-weighted prediction errors. The deficit in changing intention led to an unresolved discrepancy between the new situation and the robot’s intention, resulting in an attempt to keep waiting for the ball to come to the right hand side and an abnormal periodic action of the robot during time steps 200–300.

**Figure 3:**
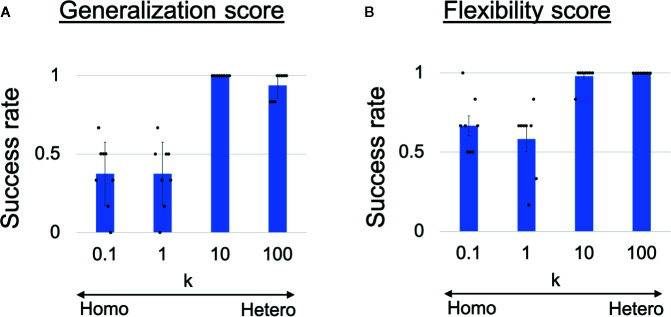
**(A)** Generalization ability and **(B)** cognitive flexibility, varying with heterogeneity of intrinsic neuronal excitability. **(A)** Success rates of generalizing learned behaviors. One-way analysis of variance indicated significant differences in success rates among the four conditions (*F*(3,28) = 38.31, *p <*0.001). Post hoc multiple comparisons using the Holm method revealed that the generalization abilities were significantly lower in the homogeneous conditions (*k* = 0.1,1) than in the heterogeneous conditions (*k* = 10,100) (all *p <*0.001). **(B)** Success rates of recognizing situation switch. One-way analysis of variance indicated significant differences in the success rates among the four conditions (*F*(3,28) = 17.59, *p <*0.001). Post hoc multiple comparisons revealed that cognitive flexibilities was significantly lower in the homogeneous conditions (*k* = 0.1,1) than in the heterogeneous conditions (*k* = 10,100) (all *p <* 0.001). Six trials were performed by each trained network (i.e., 48 trials in each *k* condition). All reported results are averages of eight trained networks and expressed as mean ± SD. “Hetero” and “homo” indicate heterogeneous and homogeneous cases, respectively.

To quantitatively analyze the observed behavioral alterations, we obtained the results of 48 trials in each heterogeneous *k* condition and the results are summarized in **Figures 3A, B**. The success rate of the ball-pass task during time steps 0–100 is shown in **Figure 3A** and was regarded as a measure of generalization ability. The performance was evaluated based on whether the robots could return the ball over the white line drawn on the workbench (**Figure 2A**). The generalization abilities were found to be significantly lower in homogeneous conditions (*k* = 0.1,1) than in heterogeneous conditions (*k* = 10,100). The success rate of recognizing a new situation is shown in **Figure 3B** and was regarded as a measure of cognitive flexibility. For the assessment of cognitive flexibility, we evaluated whether PB activity at time step 300 (the end of a trial) was appropriate (see “Evaluation of mental simulation and cognitive flexibility” in *Materials and Methods* for more details). The cognitive flexibilities were significantly lower in the homogeneous conditions (*k* = 0.1,1) than in the heterogeneous conditions (*k* = 10,100). These results suggest that robots with homogeneous networks had low cognitive flexibility and difficulty in applying learned experiences to unlearned situations (low generalization ability). However, questions remain about neural network-level characteristics underlying these behavior-level alterations and whether robots with homogeneous networks can learn the visuomotor patterns in the first place. To address these questions, a detail analysis will be given in the following parts.

### Mental Simulation and Motor Control

**Figure 4:**
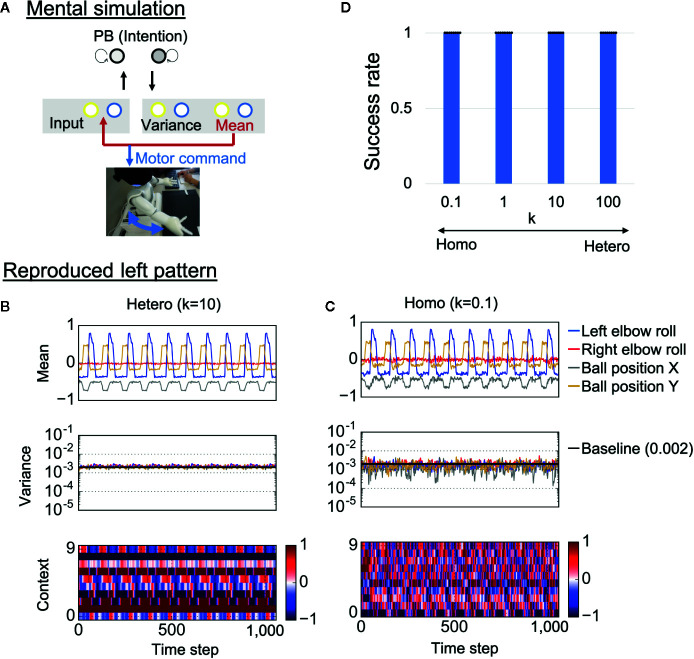
Mental simulation performance. **(A)** The robots controlled by trained networks performed mental simulation in which the neural network received previous network predictions as sensory inputs and the robot joint angles were controlled by network proprioceptive predictions. PB, parametric bias. **(B, C)** An example of generated time-series data in each condition. The robots in both the heterogeneous and the homogeneous conditions could repeatedly reproduce the learned behaviors. However, motor control, neural activity, and estimated sensory variance fluctuated in the homogeneous conditions. In addition, the estimated sensory variance tended to be lower than the variance of noise added to training data, which is described as baseline (0.002). “Mean” indicates the outputs of 4 of 10 mean neurons (mean prediction). “Variance” indicates the outputs of 4 of 10 variance neurons (estimated sensory variance). “Context” indicates the activities of 10 of 100 context neurons. **(D)** Success rate of the mental simulation in each heterogeneous condition. In all conditions, the robot could successfully reproduce learned visuomotor patterns in the mental simulation. Six trials (3 left and 3 right) were performed by each trained network (i.e., 48 trials in each *k* condition). The displayed results are averages of eight trained networks and expressed as mean ± SD. “Hetero” and “homo” indicate heterogeneous and homogeneous cases, repectively.

To confirm whether trained neural networks could successfully learn the visuomotor patterns, the robots were required to generate learned behaviors when separated from the external environment. Specifically, each trained network was required to generate visuomotor sequences by means of “closed-loop” generation, where own prediction about sensory states at a certain time step was used as the next sensory input (**Figure 4A**). Here, initial sensory inputs were taken from training data and the PB activity was set at the corresponding value. This reproduction process, where the robot’s action relies on only its internal model, can be regarded as a mental simulation. Examples of generated time-series for each condition are shown in **Figures 4B, C**. In the heterogeneous conditions, the robot’s action was smooth and the network correctly estimated the baseline of sensory variance (0.002) (**Figure 4B** and **Supplementary Video S3**). In addition, the neural activity of the context neurons clearly extracted the periodic characteristic of the target visuomotor pattern.

In the homogeneous conditions as well, the robot could repeatedly reproduce learned behavior (**Figure 4C** and **Supplementary Video S4**). However, the predicted mean and variance fluctuated, and the estimated sensory variance tended to be lower than the baseline. Due to the fluctuating proprioceptive predictions, the robot seemed to jitter. In addition, the context activity also fluctuated, and the periodicity was weak (see also frequency analysis of context activity, illustrated in **Supplementary Figure S1**). We quantitatively evaluated the performance of the mental simulation by comparing the generated time-series against training data (see “Evaluation of mental simulation and cognitive flexibility” in *Materials and Methods* for more details). **Figure 4D** shows the success rate varying with the heterogeneity of intrinsic neuronal excitability *k*. For each network condition, 48 trials were performed (3 trials were performed for each behavior in each trained network). From the graph, both homogeneous and heterogeneous networks could perfectly reproduce learned behaviors. These results suggest that low performance of homogenous networks during the real-time interaction was not caused by poor learning of the visuomotor patterns. Instead, alterations in the network process seem to be caused by overfitting to sensory noise in the learning process.

### Perception of Sensory Uncertainty

**Figure 5:**
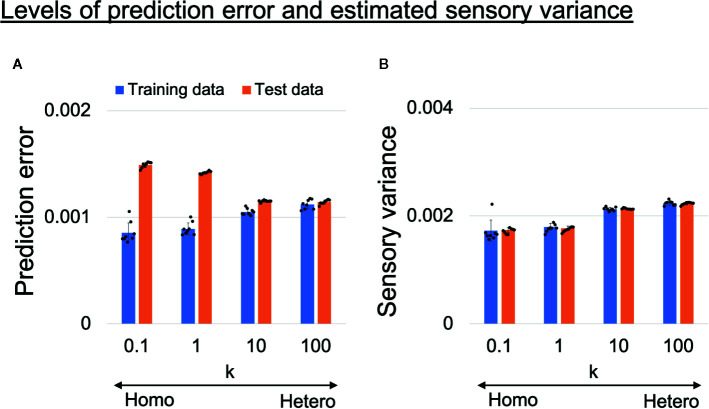
**(A)** Alterations in learning performance and **(B)** perception of sensory uncertainty. **(A)** Blue and orange bars respectively show training error and test error (error between mean prediction and target sensory state). The prediction errors were averaged over all 1,060 time steps, 10 sensory dimensions, and six input time-series data in each trained network. The difference between training error and test error became larger as the heterogeneity of intrinsic excitability *k* became smaller, suggesting overfitting to sensory noise in homogeneous networks. Two-way analysis of variance (ANOVA) indicated a significant main effect of heterogeneity *k* (*F*(3, 28) = 5.51, *p* = 0.0042) and data type (training data or test data) (*F*(1,28) = 992.45, *p <* 0.001). Regarding the main effect of heterogeneity, multiple comparisons revealed that the level of prediction error was significantly lower with *k* = 10 than with *k* = 0.1 (*p* = 0.0039) and *k* = 1 (*p* = 0.037). The two-way ANOVA also indicated a significant interaction between heterogeneity and data type (*F*(3,28) = 224.14, *p <* 0.001). To investigate the significance of the interaction between heterogeneity and data type, simple main effect analyses were performed. Significant simple main effects of heterogeneity on training error (*F*(3,28) = 31.51, *p <* 0.001) and test error (*F*(3,28) = 709.51, *p <* 0.001) were found. In addition, there was a significant simple main effect of data type for *k* = 0.1 (*F*(1,7) = 390.32, *p <* 0.001), *k* = 1 (*F*(1,7) = 733.69, *p <* 0.001), and *k* = 10 (*F*(1,7) = 109.52, *p <* 0.001). Multiple comparisons of the effect of heterogeneity on training error revealed that training errors were significantly lower in the homogeneous conditions (*k* = 0.1,1) than in heterogeneous conditions (*k* = 10,100) (all *p <* 0.001). On the other hand, test errors were significantly higher in the homogeneous conditions (*k* = 0.1,1) than in the heterogeneous conditions (*k* = 10,100) (all *p <*0.001). In addition, the test error was significantly higher when *k* = 0.1 than when *k* = 1 (*p <*0.001). **(B)** Blue and orange bars respectively show levels of estimated sensory variance for training and test data. The estimated sensory variances were averaged over 1,060 time steps, 10 sensory dimensions, and six input time-series data in each trained network. Underestimated sensory variance was observed in homogeneous networks, where the baseline of sensory variance was 0.002. Two-way ANOVA indicated a significant main effect of heterogeneity *k* (*F*(3,28) = 103.79, *p <* 0.001), but no significant main effect of data type (*F*(1,28) = 0.034, *p* = 0.86) nor interaction between heterogeneity and data type (*F*(3,28) = 0.22, *p* = 0.88). Multiple comparisons regarding the main effect of heterogeneity indicated that estimated sensory variances were significantly smaller in the homogeneous conditions (*k* = 0.1,1) than in the heterogeneous conditions (*k* = 10,100) (all *p <* 0.001). In addition, the level of estimated sensory variance with *k* = 100 was significantly larger than that with *k* = 10 (*p* = 0.011), although the difference was milder. All reported results are averages of eight trained networks and expressed as mean ± SD. “Hetero” and “homo” indicate heterogeneous and homogeneous cases, respectively.

To confirm the assumption that overfitting to training data occurred in homogeneous networks, we analyzed the prediction error and estimated sensory variance for training data and test data (unseen data not used in training). The prediction error and estimated sensory variance were calculated using generated time-series data from trained networks, where sensory inputs were obtained from training data or test data. Changes in the average levels of prediction error and estimated sensory variance are shown in **Figures 5A, B**. The values are averages of all 1,060 time steps, 10 sensory dimensions, six target datasets (3 datasets for each of left and right), and eight trained networks. In homogeneous networks (*k* = 0.1,1), the levels of the prediction error for training data were significantly smaller than those in heterogeneous networks (*k* = 10,100), but for test data the opposite was true (**Figure 5A**). These results suggest that network prediction in the homogeneous conditions was highly reliable for training data but not applicable to test data (i.e., overfitting to sensory noise was present). In addition, the estimated sensory variance in the homogeneous conditions (*k* = 0.1,1) were significantly lower than estimates in the heterogeneous conditions (*k* = 10,100) and the baseline (ground truth of 0.002) (**Figure 5B**).

### Higher-Level Neural Representation

**Figure 6:**
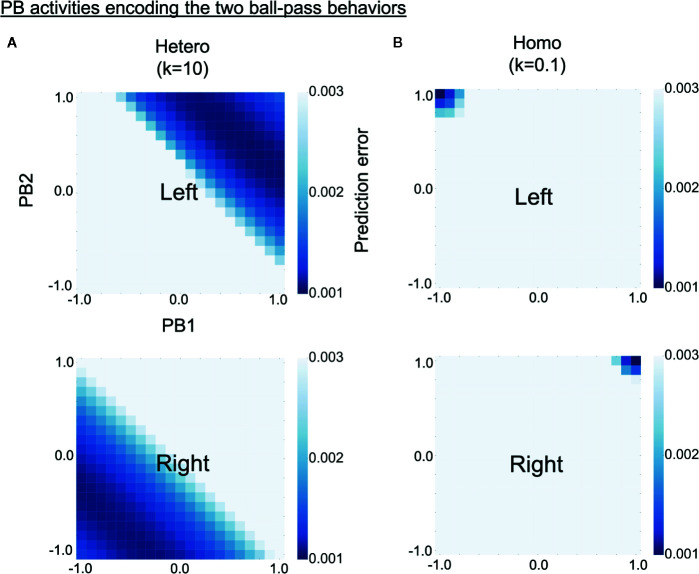
Alterations in higher-level neural representation. **(A, B)** Heat maps show levels of prediction error for training data varying with parametric bias (PB) activity (higher-level neural activity). Upper and lower panels respectively show prediction errors for training data of left and right behavior. The prediction errors are averages of three training datasets consisting of 1,060 time steps. Deep blue regions of upper heat maps represent PB activities encoding left behavior and those of lower heat maps represent PB activities encoding right behavior. Compared with the heterogeneous conditions, the homogeneous conditions were characterized by more sharply and tightly structured representations of each behavior. “PB1” and “PB2” indicate PB neurons. “Hetero” and “homo” indicate heterogeneous and homogeneous cases, respectively.

To investigate why robots controlled by homogeneous networks had problems in switching intention during the real-time interaction, we analyzed how the two learned behavioral patterns were represented in PB activity (higher-level neural activity). The S-CTRNNPB had two PB neurons, hence, the neural representation of each behavior can be shown in a two-dimensional space. Each learned behavioral pattern can be considered to be encoded in PB activities for which the prediction errors calculated using the corresponding training data are small. Heat maps in **Figure 6** show the prediction error varying with PB activity for training data of left (upper) or right (lower) behavior. The prediction errors were averaged over all three sequences consisting of 1,060 time steps. The deep blue regions in the heat maps represent the PB activities encoding the learned behaviors. As shown in the figure, left and right behaviors were represented in different PB activities for both the homogeneous and the heterogeneous conditions, indicating the difference in behaviors was recognized by the trained networks. However, in the homogeneous conditions, each behavior was very locally, or sharply, represented in the PB space. Quantitative analyses indicated larger distances between the PB activities (which encode each learned behavior) and smaller areas of the PB activities in the homogeneous condition (**Supplementary Figure S2**). The tightly organized higher-level neural representation might reflect a high confidence in the higher-level prediction (intention), which can lead to a tendency to maintain own intention in the face of environmental changes.

### Development of Synaptic Weights

**Figure 7:**
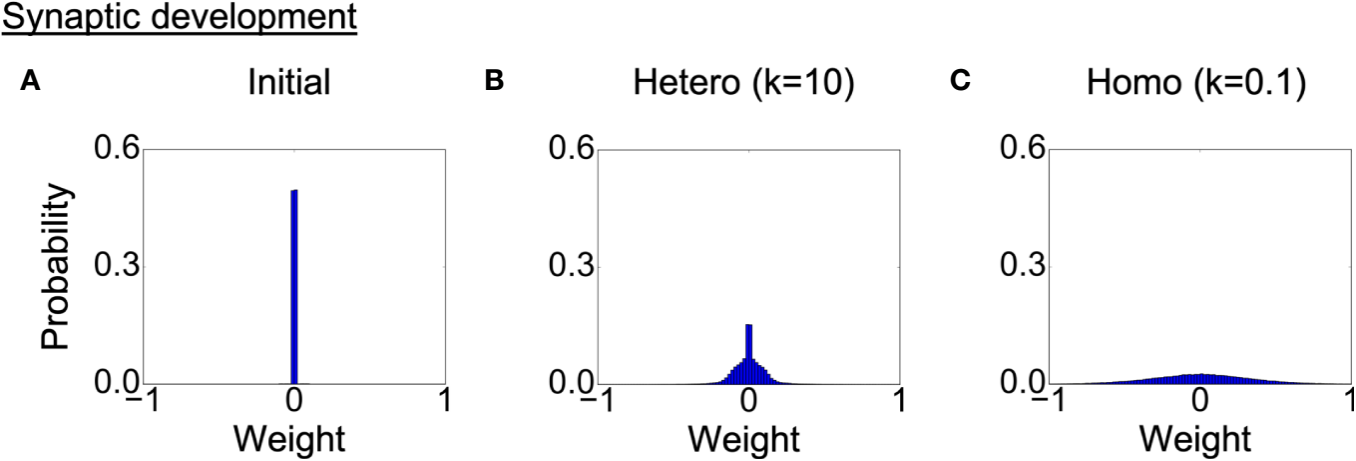
Alterations in synaptic development. **(A)** Distribution of synaptic weights before learning. The result is the average of eight networks with different initial weights. **(B, C)** Distribution of synaptic weights after learning in each condition. The result is the average of eight trained networks in each heterogeneous condition. The distribution was broader in the homogeneous conditions than in the heterogeneous conditions. This indicates that synaptic connections were overdeveloped, and there was a smaller number of weak connections. “Hetero” and “homo” indicate heterogeneous and homogeneous cases, respectively.

Finally, we analyzed the development of synaptic weights. **Figure 7A** shows the initial distribution of weights of connections between context neurons, and **Figures 7B, C** show the weight distribution after learning in each heterogeneous condition. The probabilities of the weights are averaged over eight trained networks in each condition. Because the synaptic weights in all conditions were initialized with random values following the same uniform distribution, the differences in distribution were induced solely by the differences in the heterogeneity of intrinsic neuronal excitability. The distribution of synaptic weights was broader in the homogeneous conditions than in the heterogeneous conditions, indicating the synaptic weights became excessive in the homogeneous condition. This result also suggests that there was a smaller number of weak or unwanted connections in homogeneous networks.

## Discussion

Aiming to simulate the neural, cognitive, and behavioral characteristics of a psychiatric condition, this study investigated the effects of altered heterogeneity of intrinsic neuronal excitability on learning, generalization ability, motor control, and real-time adaptation by using a humanoid robot controlled by a hierarchical RNN model. The robot first learned multiple visuomotor patterns of ball-pass behaviors based on a precision-weighted prediction error-minimization mechanism. As the result of learning, the short-term visuomotor patterns and the abstract characteristic of the different behaviors were represented in lower level (short-term) and higher-level (abstract) neural activities of the RNN. Then, the trained robot was tested *via* real-time interaction with an experimenter, during which the robots were required to generalize learned behaviors and flexibly recognize environmental changes by updating the higher-level neural activity (intention) on the basis of minimizing the precision-weighted prediction error. The robots controlled by heterogeneous networks successfully generalized learned behaviors and flexibly switched behavior in response to unpredictable environmental changes. In contrast, the robots controlled by homogeneous networks exhibited behavioral inflexibility (strong tendency to maintain own intention), difficulty in generalizing learned behaviors, and fluctuating motor control. Lines of analysis revealed that behavioral differences in homogeneous networks were caused by overfitting to sensory noise in the learning process. Due to overfitting, homogeneous networks were also characterized by underestimated sensory uncertainty (hyper sensory precision) and fluctuating neural activity. We confirmed that these changes were not observed when mean intrinsic neuronal excitability was shifted (increased or decreased) (**Supplementary Figures S3–S6**). Therefore, the observed changes are regarded as effects specific to the changes in heterogeneity of intrinsic neuronal excitability. The proposed mechanisms and observed characteristics underlying symptoms might provide an explanation that accounts for various levels of observations and theories of ASD or other neurodevelopmental disorders.

At the neural level, our results suggest a possible relationship between previous accumulated observations in neurobiology and symptoms. Previous biological studies have proposed that ASD and related neurodevelopmental disorders are associated with altered E/I balance and subsequent alterations in network excitability (7, 26). For example, most studies about ASD have suggested increased E/I ratios, partly because of the associations with epilepsy (29, 41). In fact, Rosenberg et al. (14) conducted neural network simulations suggesting perceptual characteristics of vision in ASD (e.g., a sharper gradient of attention) could be induced by decreased inhibition (an increased E/I ratio). However, there are other studies that have reported decreased E/I ratios in ASD (42, 43). A recent article reviewing empirical studies pointed out that the direction of E/I imbalance associated with the condition is not clear (32). Furthermore, both excitation and inhibition can be altered through homeostatic plasticity (i.e., primary deficits in excitation or inhibition may induce secondary compensatory changes in the other) (33). As an alternative to an increased or decreased E/I ratio model, this study simulated differences in the heterogeneity of intrinsic neuronal excitability. Heterogeneity of intrinsic neuronal excitability is the neuron-to-neuron variability of intrinsic excitability, which may be controlled by synaptic and channel functions regulating neural excitability that has been proposed to be different in neurodevelopmental disorders. The reason for focusing on this characteristic of neural excitability is that it is considered to be important for efficient coding (34–36), and a part of psychiatric symptoms could be regarded as resulting from disruptions in efficient coding of sensory information (overfitting to sensory noise) (23, 44). Indeed, our results show that homogeneous networks with low levels of variance in intrinsic neuronal excitability (*k* = 0.1,1) led to overfitting to sensory noise, whereas heterogeneous networks with high levels of variance (*k* = 10,100) exhibited good performances in behavioral flexibility and generalization ability. Although it may be difficult to directly connect the levels of the variance in neuronal excitability in our abstract-level model and the real neural system, the difference in the variance settings of homogeneous networks and heterogeneous networks can be regarded as roughly reasonable based on previous studies. For example, the variance in firing threshold found in biological neurons is typically about 10*mV* ^2^ but is highly variable, such that it can range from approximately one hundredth to 10-fold of the typical value (39, 40, 45). The level of variance in neuronal excitability deviating from this range may be considered to result in abnormal network functions, as our results suggest. Furthermore, a previous numerical study (34) using a spiking neural network model showed that the coding efficiency was lowest when the variance in firing threshold was around 0*mV* ^2^ and highest when the variance was approximately 16*mV* ^2^, which is within the range of the variance in normal heterogeneous settings in our study. Our findings on the association between the neural-level characteristic and the set of multifaceted behavioral alterations might have implications for underlying mechanisms of neurodevelopmental disorders. We focus in particular on temporal coding and processing, where sensory information is essentially encoded in temporal patterns of neuronal activities (not activity levels themselves). Thus, although excitatory and inhibitory circuits are not explicitly considered in the utilized neural network model, the results obtained in this study might have general implications for dynamic neural systems.

Our results might provide insights into alterations in synaptic development and neural activity in neurodevelopmental disorders. In terms of network development, psychiatric disorders are thought to be associated with alterations in pruning, the process of eliminating excess synaptic connections (46). For example, excessive synapses due to deficits in pruning were observed in postmortem brains from human subjects with ASD (47), and synapse elimination was observed to be increased in individuals with schizophrenia (48). In the present study, homogeneous networks induced excessive development of synaptic weights, and the number of weak or unwanted synaptic connections was lower than in heterogeneous networks. This suggests that the number of synaptic connections eliminated *via* pruning might be lower in homogeneous networks. In addition, functional magnetic resonance imaging studies have suggested that neural responses in ASD are unreliable and noisy (49, 50), which is consistent with our experimental results showing fluctuating neural activity in homogeneous networks. These findings about the characteristics of neural networks provide potential targets for future studies, such as evaluation of associations between these network-level characteristics and symptoms in human subjects and animal models.

Overall, this study suggests heterogeneity of intrinsic neuronal excitability is important for network function and efficient temporal coding. Many techniques have been proposed to realize robust network functions by avoiding overfitting in machine learning, including neural networks. A simple technique is to use a variety of training data (e.g., data augmentation) to attenuate over-specificity to a specific training data and increase generalization ability. A recent perceptual learning study has shown that inflexibility in individuals with ASD might be attributable to over-specificity and could be significantly attenuated by adding a dummy trial in the learning phase (51). This finding might support our results suggesting that inflexibility in ASD may be associated with overfitting to training data. In addition, reducing the number of non-negligible synaptic weights is also effective for avoiding overfitting. A representative example is the “weight decay” method, which introduces weight penalties that drive synaptic weights to have smaller magnitude (52). Another method, “dropout,” stochastically thins the number of synaptic weights to reduce overfitting (53). In our experiment, synaptic weights remained at low values in heterogeneous networks but developed excessively in homogeneous networks, indicating the same outcome can also be realized by controlling neuron-to-neuron variability of intrinsic excitability. This result shows that important characteristics underlying efficient coding (e.g., synaptic weight values and heterogeneity of excitability) may be mutually linked. In addition, the proposed condition induced by the disruption of efficient coding could be generated from either a defect in controlling weight values (e.g., a defect in synaptic pruning) or altered heterogeneity of neuronal excitability.

At the cognitive level, our findings using a predictive coding framework may extend the computational understanding of psychiatric symptoms. Previous computational theories have posited that aberrant precision-weighting of prediction error is associated with psychiatric disorders (22–25). In particular, symptoms of ASD and related neurodevelopmental disorders have been explained as excessively high precision-weighting of sensory prediction error (21–23, 44). However, within a framework in which precision-weighted prediction error causes the brain to update its prediction, how can insistence on sameness or behavioral inflexibility (tendency to maintain own prediction), one of the typical characteristics of ASD, be explained? The discrepancy could be explained by considering hierarchical representations shaped by overfitting to sensory noise and discriminating between learning and real-time adaptation. Overfitting to sensory noise observed in homogeneous networks shaped with fluctuating strong predictions, which was very reliable for learned data but not for unlearned situations. In addition, the effects of overfitting affected the higher level of the hierarchy representing different types of visuomotor patterns. As the result, each behavioral pattern was very locally and sharply represented in the higher-level neural activity. The tightly organized higher-level representation might be considered to cause strong confidence in own intention (higher-level prediction) and a tendency to maintain the intention during real-time adaptation. The co-occurrence of under-estimated sensory uncertainty (hyper sensory precision) and strong predictions might also have implications for the relationship between ASD and schizophrenia because hallucinations, which is a hallmark symptom of schizophrenia, have been proposed to be associated with strong prior beliefs (25). Furthermore, not only underestimated sensory uncertainty but also increased variability of estimated sensory uncertainty was observed in homogeneous networks. The unusual modulations of estimated sensory uncertainty might explain the heterogeneous characteristics of symptoms of ASD (e.g., the coexistence of hypersensitivities and hyposensitivities) as described in a recent study (54), and might provide additional insight into an empirical study showing adults with ASD overestimate the volatility of sensory environments (55).

In summary, our findings might explain the relationships among neural, cognitive, and behavioral characteristics of ASD or other neurodevelopmental disorders. Our results suggest that perceptual or sensorimotor-level symptoms (e.g., reduced generalization, and motor clumsiness) may result from overfitting to sensory noise during learning while behavioral inflexibility may be associated with strong confidence in own higher-level prediction, which has been shaped by the overfitting. Other than inflexibility, reduced generalization, and motor clumsiness, the proposed model might also be able to explain unusual sensory sensitivity in ASD as unusual sensory precision. Given the consistency with the neural-level data (e.g., variability in neural activity and synaptic development) and the computational-level understanding, as well as the various behavior-level observations, our model might capture the characteristics of ASD in particular. A key point is that we observed the multifaceted behavioral symptoms in a physical robot interacting with an unpredictably changing real environment. Although few computational modeling studies of psychiatric disorders have used a physical robot, the robotic approach might be useful to understand symptoms emerging from the interaction among the neural network, body, and environment, which are analogs of essential components forming human behavior (16–18). Because psychiatric disorders partly share symptoms, the mechanism that we have proposed may have implications for various psychiatric conditions, such as schizophrenia, obsessive compulsive disorder, and attention deficit hyperactivity disorder (5, 6). The produced hypothesis about the mechanism may be testable by investigating the relationships among neural, cognitive, and behavioral characteristics in human subjects and animal models. Such a comprehensive assessment of patients might contribute to the establishment of precise diagnostic categories and an approach for treatment that accounts for the characteristics of each person (“precision psychiatry”) (56, 57). In addition, a computational understanding of mechanisms underlying psychiatric symptoms might help clinicians, patients, and their relatives to strictly establish a concept of symptoms and the presence of those symptoms (44). A limitation of this study is that we investigated the effect of heterogeneity of only neuronal excitability but there may be other ways of modeling altered neural heterogeneity that may affect efficient coding, such as manipulations of neuronal gain and firing intensity (58, 59). Comparing the effects of these parameters will be important for understanding individual differences in psychiatric patients. Furthermore, whether the proposed model can explain the deficits in language and social interaction in ASD is also an important issue. Future study involving quantitative comparisons between robot models and human subjects on the same basis in terms of behaviors and brain activities can be expected to contribute to finer understanding of psychiatric disorders by combining computational and clinical studies.

## Materials and Methods

### Neural Network Model

The current S-CTRNNPB model is a type of continuous-time RNN (CTRNN). A CTRNN implements a feature of biological neurons in the sense that neuronal activities are determined by the past history of neural states as well as current synaptic inputs (60, 61). The neuronal model is based on the conventional firing rate model. In the firing rate model, it is assumed that the essential information is carried by the mean firing rate in a given time interval, and the relation between the output of a neuron and the internal state is described by a sigmoid function, which models the saturation of the firing rate. The current model cannot consider consistency in physiological details, such as features of individual spikes and characteristics of individual synapses. Therefore, the results obtained in this study can be discussed at the macro level as mechanisms of biological neural systems.

#### Forward Prediction

The internal state of the *i*th neuron at time step *t*, notated $u_{t,i}^{\left(s\right)}(t\geq 1)$, is calculated as

(1) $$u_{t,i}^{\left(s\right)}=\{\begin{array}{l} u_{t-1,i}^{\left(s\right)}\;(i\in I_{P}),\\ \begin{array}{l} \frac{1}{\tau_{i}}\left(\sum_{j\in I_{I}}w_{ij}x_{t,j}^{\left(s\right)}+\sum_{j\in I_{C}}w_{ij}c_{t-1,j}^{\left(s\right)}+\sum_{j\in I_{P}}w_{ij}p_{t,j}^{\left(s\right)}+b_{i}\right)\\ +\left(1-\frac{1}{\tau_{i}}\right)u_{t-1,i}^{\left(s\right)}\;(i\in I_{C}), \end{array}\\ \sum_{j\in I_{C}}w_{ij}c_{t,j}^{\left(s\right)}+b_{i}\;(i\in I_{M},I_{V}), \end{array}$$

where *I*_I_, *I*_M_, *I*_V_ *I*_C_, and *I*_P_ are index sets of the input, mean, variance, context, and PB neurons, respectively; *w_ij_*is the weight of the synaptic connection from the *j*th neuron to the *i*th neuron; $x_{t,j}^{\left(s\right)}$ is the *j*th input at time step *t* of the *s*th sequence; $c_{t,j}^{\left(s\right)}$ is the *j*th context activity; $p_{t,j}^{\left(s\right)}$ is the *j*th PB activity; *τ_i_*is the time constant of the *i*th neuron; and *b_i_*is the bias of the *i*th neuron, which determines the intrinsic neuronal excitability. From the equation above, PB neurons can be regarded as a specific type of context neurons whose time constant is infinite. In this study, we set the initial values of the internal states of the context neurons to zero, and those of the PB neurons are optimized for each target temporal sequence of learning. This indicates that differences among multiple target temporal sequences are represented in the activities of PB neurons and dynamics of context activities. The output of each neuron is calculated using the following activation functions:

(2) $$\begin{array}{cc} p\begin{array}{c} \left(s\right)\\ t,i \end{array}=\tanh\;\left(u\begin{array}{c} \left(s\right)\\ t,i \end{array}\right) & (0\leq \end{array}t\wedge i\in I_{\text{P}}),$$

(3) $$\begin{array}{cc} c\begin{array}{c} \left(s\right)\\ t,i \end{array}=\tanh\;\left(u\begin{array}{c} \left(s\right)\\ t,i \end{array}\right) & (0\leq \end{array}t\wedge i\in I_{\text{C}}),$$

(4) $$\begin{array}{cc} y\begin{array}{c} \left(s\right)\\ t,i \end{array}=\tanh\;\left(u\begin{array}{c} \left(s\right)\\ t,i \end{array}\right) & (1\leq \end{array}t\wedge i\in I_{\text{M}}),$$

(5) $$\begin{array}{cc} v\begin{array}{c} \left(s\right)\\ t,i \end{array}=\exp\;\left(u\begin{array}{c} \left(s\right)\\ t,i \end{array}\right) & (1\leq \end{array}t\wedge i\in I_{\text{V}}).$$

#### Parameter Optimization

The S-CTRNNPB performs parameter optimization *via* the gradient descent method, aiming to minimizing the negative log-likelihood. The negative log-likelihood is formally given as

(6) $$L\begin{array}{c} \left(s\right)\\ t,i \end{array}=\frac{\ln\left(2\pi v\begin{array}{c} \left(s\right)\\ t,i \end{array}\right)}{2}+\frac{{\left(\hat{y}\begin{array}{c} \left(s\right)\\ t,i \end{array}-y\begin{array}{c} \left(s\right)\\ t,i \end{array}\right)}^{2}}{2v\begin{array}{c} \left(s\right)\\ t,i \end{array}}.$$

Here, ${\hat{y}}_{t,i}^{\left(s\right)}$ is the target value of the *i*th mean neuron corresponding to the *s*th sequence. Minimizing this negative log-likelihood can be regarded as minimizing the precision-weighted (inverse variance-weighted) prediction error. Therefore, in this study, the negative log-likelihood is referred to as the precision-weighted prediction error.

During learning, parameters—including synaptic weights *w_ij_*, biases *b_i_*of mean and variance neurons, and initial internal states of PB neurons $u_{0,i}^{\left(s\right)}\;(i\in I_{P})$ —are updated, and the biases *b_i_*of context neurons are not updated in order to fix the distribution. Parameter optimization is performed by minimizing the sum of the negative log-likelihood over all sensory dimensions, time steps, and sequences as

(7) $$L=\sum_{s\in I_{\text{S}}}\sum_{t=1}^{T^{\left(s\right)}}\sum_{i\in I_{\text{M}}}L\begin{array}{c} \left(s\right)\\ t,i \end{array},$$

where *I*_S_ and *T* ^(^ *^s^* ^)^ represent the index set and the length of the *s*th target temporal sequence, respectively. The partial derivative of each parameter, (*∂L*)*/*(*∂θ*), can be solved using the back-propagation-through-time method (37, 62).

During real-time interaction after learning, only the internal states of the PB neurons are updated; other parameters are fixed. In this phase, the negative log-likelihood within a short time window *W* is accumulated as

(8) $$L=\sum_{t'=t-W+1}^{t}\sum_{i\in I_{\text{M}}}L\begin{array}{c} \left(s\right)\\ t',i \end{array}.$$

The time window slides with the increment of the network time step *t*. Using the accumulated negative log-likelihood, the internal states of the PB neurons at time step *t*−*W* are optimized. The partial derivative of the internal states of PB neurons is also calculated by the back-propagation-through-time algorithm.

In both the learning and real-time interaction phases, parameters permitted to be optimized are collected by *θ*, and *θ* at the *n*th epoch is updated by gradient descent on the accumulated negative log-likelihood *L*:

(9) θ (n)=θ (n-1)+Δθ (n),

(10) $$\Delta \theta \;(n)=-\alpha \frac{\partial L}{\partial \theta }+\eta \Delta \theta \;(n-1).$$

Here, *α* is the learning rate and *η* is a coefficient representing the momentum term. In this study, *α* and *η* are set at 0.0001 and 0.9, respectively.

#### Experimental Environment

We used a small humanoid robot, NAO T14 (SoftBank Robotics, Paris, France), that has a body corresponding to only the upper half of the human body. The robot was placed in front of a workbench and carried out a ball-playing interaction with an experimenter standing at the opposite side. The robot’s action involved only movements of the arms, with 4 degrees of freedom for each arm (2 degrees for shoulders and 2 for elbows). In addition, a camera installed in the robot’s mouth obtained the center of gravity coordinates for the yellow object, which corresponds to the two-dimensional visual inputs for ball position. Using the minimum and maximum values of each sensory input, the values of joint angles and the ball position were normalized to values ranging from −0.8 to 0.8. During task execution, the robot received the sensory states every 100 ms. The dimensions of the workbench and diameter of the ball were approximately 45 × 5 × 30 cm and 9 cm, respectively. White lines were drawn on both ends of the workbench at 20 cm from the robot, and they were used to evaluate the performance of the ball-passing task in the real-time interaction experiment. The coordinates of the inner end of the white lines as captured by the robot’s camera were (−0.40,0.16) (left line) and (0.40,0.16) (right line) (**Figure 2A**).

### Training

The neural network was trained by predictive learning using target perceptual sequences recorded in advance. The target sequences were recorded while the robot repeatedly performed each ball-pass behavior, where the arm movement was generated exactly by following preprogrammed trajectories instead of the ones generated by the neural network model. Each sequence of the two behavioral patterns was obtained as a sequence of 10-dimensional vectors (8-dimensional proprioception of joint angles and two-dimensional vision sense of ball position). In the experiment, six sequences were prepared for each behavioral pattern, and the time lengths of the sequences were 1,060 time steps (10 cycles) for both right and left behaviors. Three sequences of each behavior were used as training data and the others were used as test data. The neural network learned to reproduce the sequences of training data. The objective of learning was to find the optimal values of the parameters (synaptic weights and internal states of PB neurons) by minimizing negative log-likelihood or, equivalently, the precision-weighted prediction error. Initially, the network produced random sequences with randomly initialized parameters. The parameters were updated toward minimizing the negative log-likelihood accumulated through the time length of the target sequences. After repeating the update process many times, the network began to produce visuomotor sequences with the same stochastic properties as the training data. In addition, the associations between a particular pattern of target sequence and specific internal states of PB neurons were self-organized.

### Real-Time Adaptation

In the real-time interaction phase, the robot’s intention (PB activity) was first set at a certain state corresponding to a learned behavior and the robot performed the corresponding interaction with the experimenter. Then, the situation (ball position) was unpredictably changed by the experimenter. The goal of the robot was to flexibly recognize the environmental changes using visual observations. The real-time adaptation process during the task execution of the robot was performed based on an interaction between top-down prediction generation and bottom-up parameter modulation. In the top-down prediction generation process, the network generated a temporal sequence corresponding to time steps from *t*−*W* +1 to *t*, based on the sensory inputs at time step *t* −*W* + 1 and the constant PB activity (intention). The visuomotor sequence was generated by a “closed-loop” process, using the prediction of mean values of sensory states at each time step as inputs to the next time step. The initial inputs for joint angles at time step *t* −*W* + 1 are the generated mean predictions at time step *t* −*W*, and the vision states characterize the vision data captured by the camera at time step *t* −*W* + 1. In the bottom-up modulation process, the precision-weighted prediction error at each time step within time window *W* was calculated from the vision-state predictions, variance, and actual observation (see **Figure 2A**). The PB activity (intention) was updated in the direction of minimizing the accumulated precision-weighted prediction error. A temporal sequence within the time window was re-generated from the updated PB activity. After repeating these top-down and bottom-up processes for a certain duration, the network generated predictions for time step *t* + 1; the predicted joint angles were sent to the robot as the target for the next joint positions. This procedure, in which the recognition and prediction in the past are reconstructed from the current sensory information, is a “postdiction” process (63, 64), and the predictions generated for time steps from *t*−*W* +1 to *t* are “postdictions” from the past, rather than literal predictions.

### Parameter Settings for the Experiments

The numbers of input, mean, and variance neurons were *N*_I_ = *N*_M_ = *N*_V_ = 10, respectively, corresponding to the dimension of the robot’s sensory states, and the number of PB neurons was *N*_P_ = 2. The number and time constant of context neurons were *N*_C_ = 100 and *τ_i_*= 4, respectively. For learning, the weights of the synaptic connections *w_ij_*(*j* ∈ *I*_I_,*I*_C_) were initialized with random values that follow uniform distributions on the intervals $\left[-\frac{1}{N_{I}},\frac{1}{N_{I}}]\;(j\in I_{I}\right)$ and $\left[-\frac{1}{N_{C}},\frac{1}{N_{C}}]\;(j\in I_{C}\right)$. Biases of the mean and variance neurons *b_i_*(*i* ∈ *I*_M_,*I*_V_) were initialized with random values following a uniform distribution on the intervals [−1,1], and the internal states of PB neurons were initialized to 0. These parameters were updated 300,000 times during learning. Biases *b_i_*of the context neurons were initialized with and fixed to random values following a Gaussian distribution *N*(0*,k*) (*k* ∈{0.1,1,10,100}). In the real-time adaptation process, the internal states of the PB neurons were updated 50 times at each time step for a time window of length *W* = 10.

### Evaluation of Mental Simulation and Cognitive Flexibility

In the mental simulation experiment, trained networks generated temporal sequences by the “closed-loop” process and the robot’s joint angles were controlled based on the mean proprioceptive predictions. To judge whether the robot’s action during the mental simulation is appropriate, the generated time-series of sensory states was compared with the target (learned) data. A simple way to compare two time-series is to calculate the distances between values at corresponding time steps within a certain time window. However, this method is not necessarily appropriate for comparing the general characteristics of time-series because a phase shift will increase the distance between the series, even when the series is appropriate. To address this, our study characterized the visuomotor sequences as probability distributions of sensory states by compressing time-series data in the time axis direction and used the Kullback–Leibler (KL) divergence for comparison. In the evaluation, the probability distribution of each sensory state was found, with the one-dimensional space (−1,1) divided into *N*_bin_ = 50 subspaces. Using the acquired probability distributions, we calculated the KL divergence between the distribution of a certain sensory state in the mental simulation and the distribution of the corresponding sensory state in target data for each sensory state. Then, the sum of the KL divergences over all sensory states was calculated and this was regarded as a similarity score. The robot’s action was judged as correct if the similarity score was less than a threshold *ξ*, set here as 0.5 times the minimum value of similarity scores between each pair of training data.

(11) $$SS(p||q)=\sum_{k\in I_{{\text{M}}_{\text{joint}}}}D_{KL}(p_{y_{k}}||q_{y_{k}})$$

(12) $$SS(p||q)<\xi =0.5\times \min_{q_{i}\in U_{L},q_{j}\in U_{R}\vee q_{i}\in U_{R},q_{j}\in U_{L}}SS(q_{i}||q_{j})$$

Here, *SS(p||q)* is the similarity score between the probability distribution of sensory states in the experiment *p* and the probability distribution in target data *q*; *U_L_*is a set of the probability distributions of sensory states in training data of left behavior; and *U_R_*is that of right behavior.

The above methods were also used for the evaluation of cognitive flexibility in the real-time interaction experiment. In this case, the networks were required to generate temporal sequences by the “closed-loop” method using PB activity (intention) at the end of each trial (time step 300). The idea is that if the neural network successfully recognized the environmental change, the time-series generated by the network with the PB activity at time step 300 is expected to be similar to the target time-series of the new situation. The success rates were calculated by comparing the time-series generated by the networks with the target sequences (training data), as with the mental simulation.

### Statistical Analysis

In the analysis of prediction error and estimated sensory variance for training data or test data, two-way analysis of variance (ANOVA) was used. In other statistical analyses, we used one-way ANOVA followed by non-paired Holm tests. All statistical tests were two-tailed and the significance level was set at *p <*0.05. No statistical methods were used to predetermine sample size. Data analysis was conducted using R software (version 3.3.2).

## Data Availability Statement

All datasets presented in this study are included in the article/**Supplementary Material**.

## Author Contributions

HI conceived the study, performed the experiment, and analyzed the data. HI, SM, YY, and TO designed the experiment and analysis, and wrote the paper.

## Funding

This work was supported by a MEXT Grant-in-Aid for Scientific Research on Innovative Areas, “Constructive Developmental Science” (JP24119003), a JSPS Grant-in-Aid for JSPS Research Fellow (No. JP19J20281), JSPS KAKENHI grants (Nos. JP17K12754, JP18KT0021, and JP19H04998), JST CREST grants (Nos. JPMJCR16E2 and JPMJCR15E3), and the Program for Leading Graduate Schools, “Graduate Program for Embodiment Informatics” of the Ministry of Education, Culture, Sports, Science and Technology, Japan.

## Conflict of Interest

The authors declare that the research was conducted in the absence of any commercial or financial relationships that could be construed as a potential conflict of interest.
