# Supplementary material for: Homogeneous Intrinsic Neuronal Excitability Induces Overfitting to Sensory Noise: A Robot Model of Neurodevelopmental Disorder
Source: Front Psychiatry. 2020 Aug 12;11:762. doi: 10.3389/fpsyt.2020.00762 (PMC7434834; doi:10.3389/fpsyt.2020.00762)
Supplement: Supplementary file 1 [file DataSheet_1.pdf]

Supplementary Material

1 SUPPLEMENTARY FIGURES

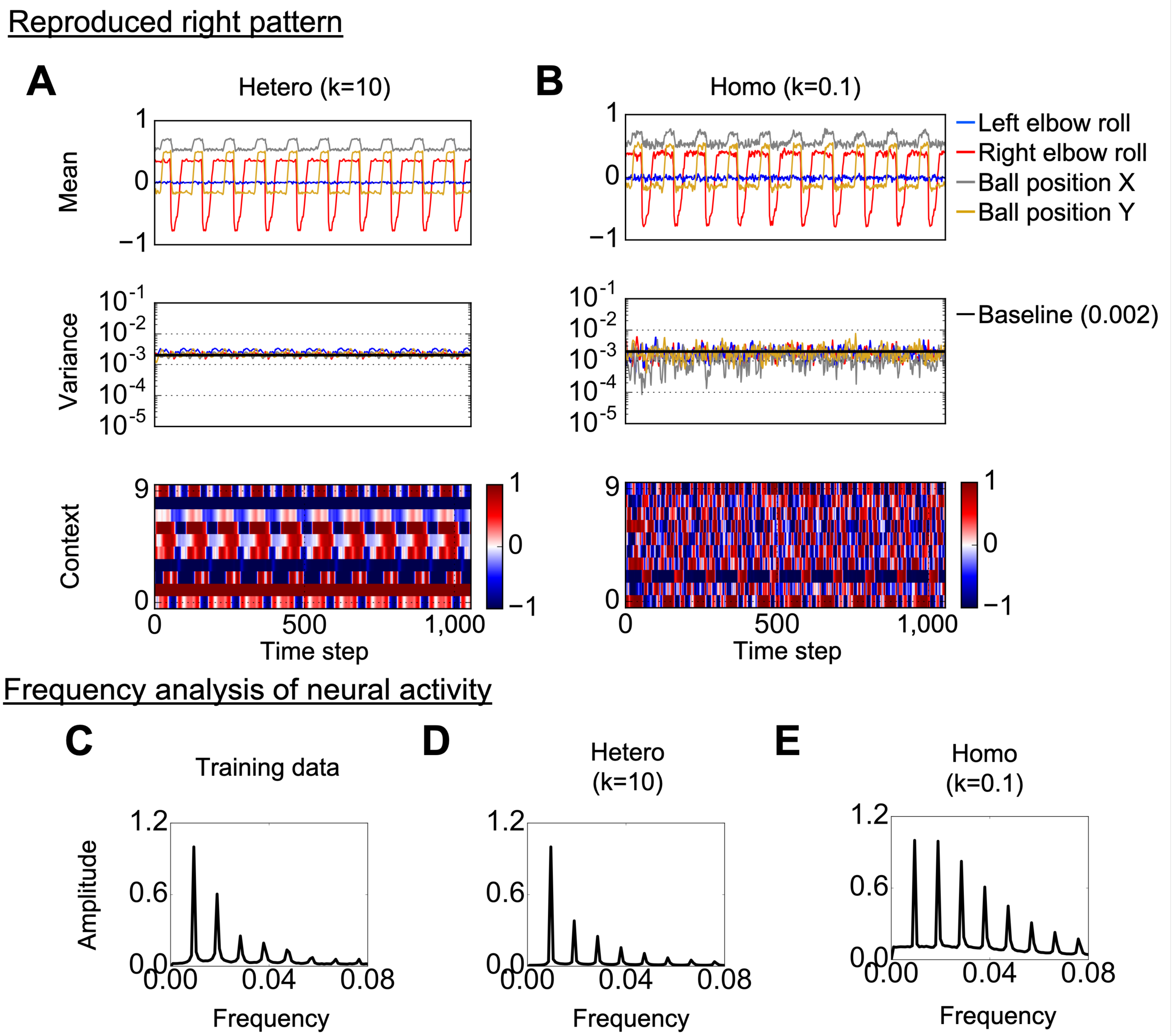

Figure S1. Learning results and frequency analysis of neural activity. (**A, B**) An example of time-series data generated by a trained neural network in each heterogeneous condition, where training data were used as sensory inputs. In the homogeneous conditions, fluctuating mean predictions and neural activity were observed. Variance predictions also fluctuated and tended to be lower than the baseline of sensory variance (0.002) indicated by the black line. "Mean" indicates outputs of 4 of 10 mean neurons. "Variance" indicates outputs of 4 of 10 variance neurons. "Context" indicates activities of 10 out of 100 context neurons. (**C**) Result of the Fourier transform (FT) of training data. The result is the average of all 10 sensory dimensions and 6 training datasets (3 for left behavior and 3 for right behavior). (**D, E**) Result of the FT of context activity in the reproduction process of training data in each heterogeneous condition. The results are averages of all 100 context neurons, 6 training datasets, and 8 trained networks. The amplitudes were mapped to values ranging from 0.0 to 1.0, normalized by the maximum value. In the heterogeneous conditions, the frequency component of about 0.01 and its harmonic components were salient, and other frequency components were very weak. This indicates heterogeneous networks captured the periodic characteristic of the training data. In the homogeneous conditions, however, the frequency components other than the harmonic components were relatively strong, suggesting the neural activity was unreliable. "Hetero" and "homo" indicate, respectively, heterogeneous and homogeneous cases.

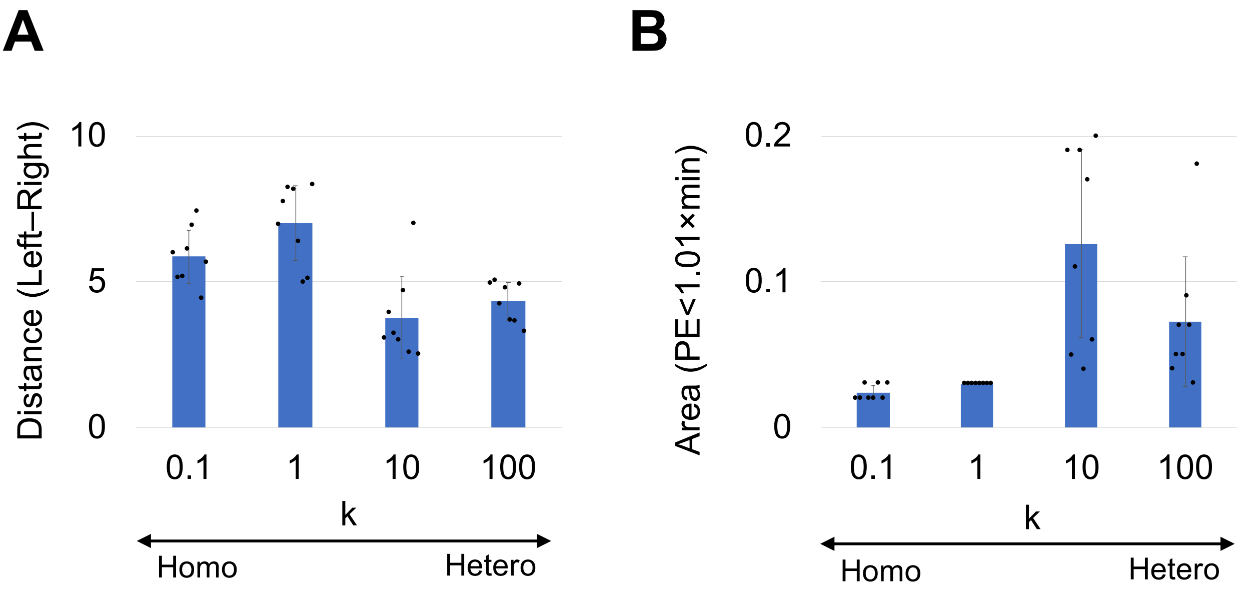

Figure S2. Association of learned behaviors in higher-level neural representation. (**A**) Distances between internal states of PB neurons corresponding to each behavior. The distances were calculated using mean internal PB states acquired for 3 training datasets for each behavioral pattern. The calculated distances in homogeneous networks ($k = 0.1, 1$) were larger than those in heterogeneous networks ($k = 10, 100$). One-way ANOVA indicated a significant difference among the four network conditions ($F(3, 28) = 12.43$, $p < 0.001$). Post hoc multiple comparisons using the Holm method indicated significant differences from $k = 10$ in the conditions $k = 0.1$ ($p = 0.0053$) and $k = 1$ ($p < 0.001$), but not in $k = 100$ ($p = 0.34$). (**B**) Areas of PB activities at which prediction errors (PEs) for training data were lower than 1.01 times the minimum value of the PE, varying with PB activity. The calculated areas in homogeneous networks were smaller than those in heterogeneous networks. One-way ANOVA indicated a significant difference among the four conditions ($F(3, 28) = 10.17$, $p < 0.001$). Post hoc Holm testing indicated significant differences from $k = 10$ in conditions $k = 0.1$ ($p < 0.001$) and $k = 1$ ($p < 0.001$), but not in $k = 100$ ($p = 0.064$). The larger distances and smaller areas of PB activities encoding two learned behaviors in homogeneous networks suggest reduced generalization in the higher-level neural representation. All results are averages of 8 trained networks and expressed as mean ± SD. “Hetero” and “homo” indicate, respectively, heterogeneous and homogeneous cases.

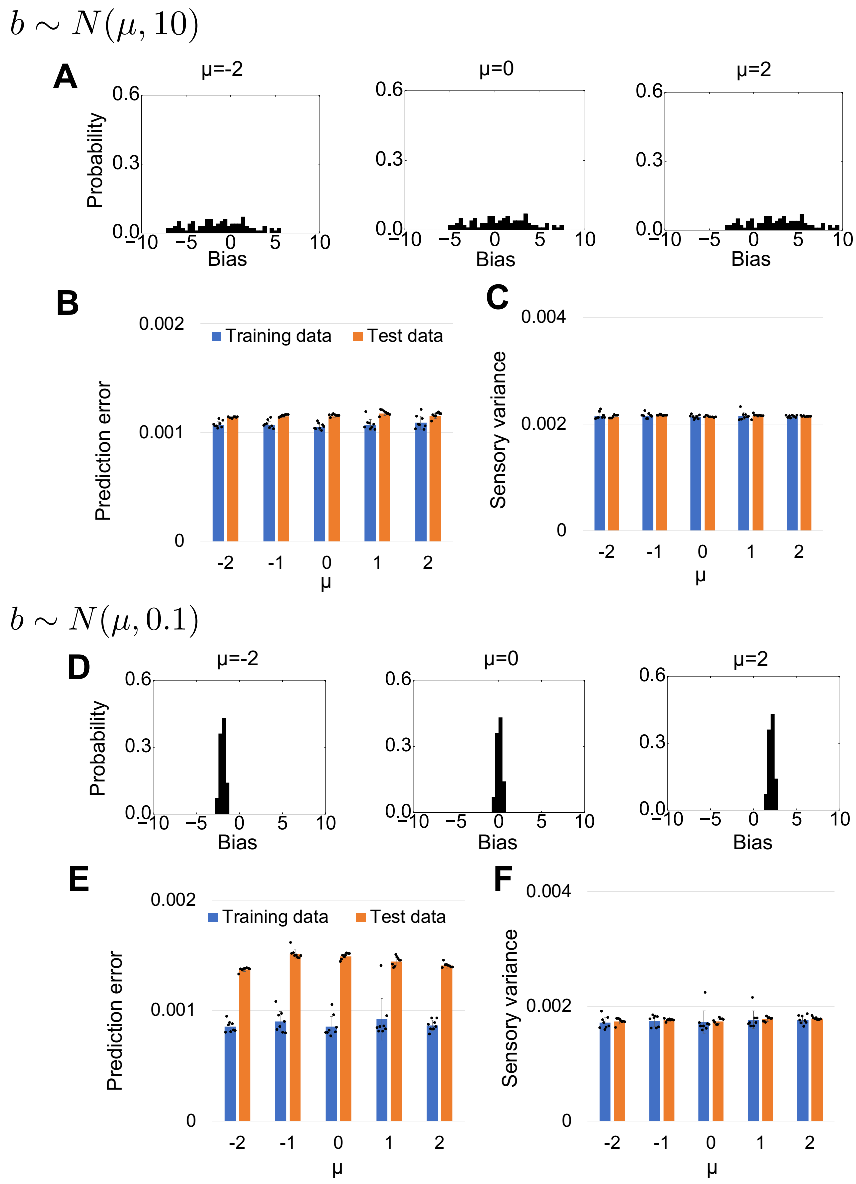

Figure S3. No significant effects of shifts of mean neuronal excitability on learning performance. (**A**) Distribution of bias b, (**B**) level of prediction error, and (**C**) level of estimated sensory variance in a heterogeneous condition ($k = 10$) varying with mean excitability μ. As in Figure 5, the prediction errors and estimated sensory variances were averaged over all 1060 time steps, 10 sensory dimensions, and 6 input time-series datasets in each trained network. Two-way ANOVA failed to indicate a significant effect of mean excitability on either the prediction error and estimated sensory variance (for prediction error, main effect of μ [$F(4, 35) = 0.39$, $p < 0.82$], main effect of data type [$F(1, 35) = 106.67$, $p < 0.001$], and interaction [$F(4, 35) = 1.98$, $p = 0.12$]; for estimated sensory variance, main effect of μ [$F(4, 35) = 0.78$, $p = 0.54$], main effect of data type [$F(1, 35) = 0.0013$, $p = 0.97$], and interaction [$F(4, 35) = 0.40$, $p = 0.81$]). (**D**) Distribution of bias b, (**E**) level of prediction error, and (**F**) level of estimated sensory variance in a homogeneous condition ($k = 0.1$) varying with mean excitability μ. The prediction errors and estimated sensory variances are averages of all 1060 time steps, 10 sensory dimensions, and 6 input time-series datasets in each trained network. Two-way ANOVA again failed to indicate a significant effect of mean excitability on either the prediction error or estimated sensory variance (for prediction error, main effect of μ [$F(4, 35) = 2.46$, $p = 0.064$], main effect of data type [$F(1, 35) = 1220.46$, $p < 0.001$], and interaction [$F(4, 35) = 2.33$, $p = 0.075$]; for estimated sensory variance, main effect of μ [$F(4, 35) = 0.55$, $p = 0.70$], main effect of data type [$F(1, 35) = 0.79$, $p = 0.38$], and interaction [$F(4, 35) = 0.052$, $p = 0.99$]). All results are averages of 8 trained networks and expressed as mean $\pm$ SD.

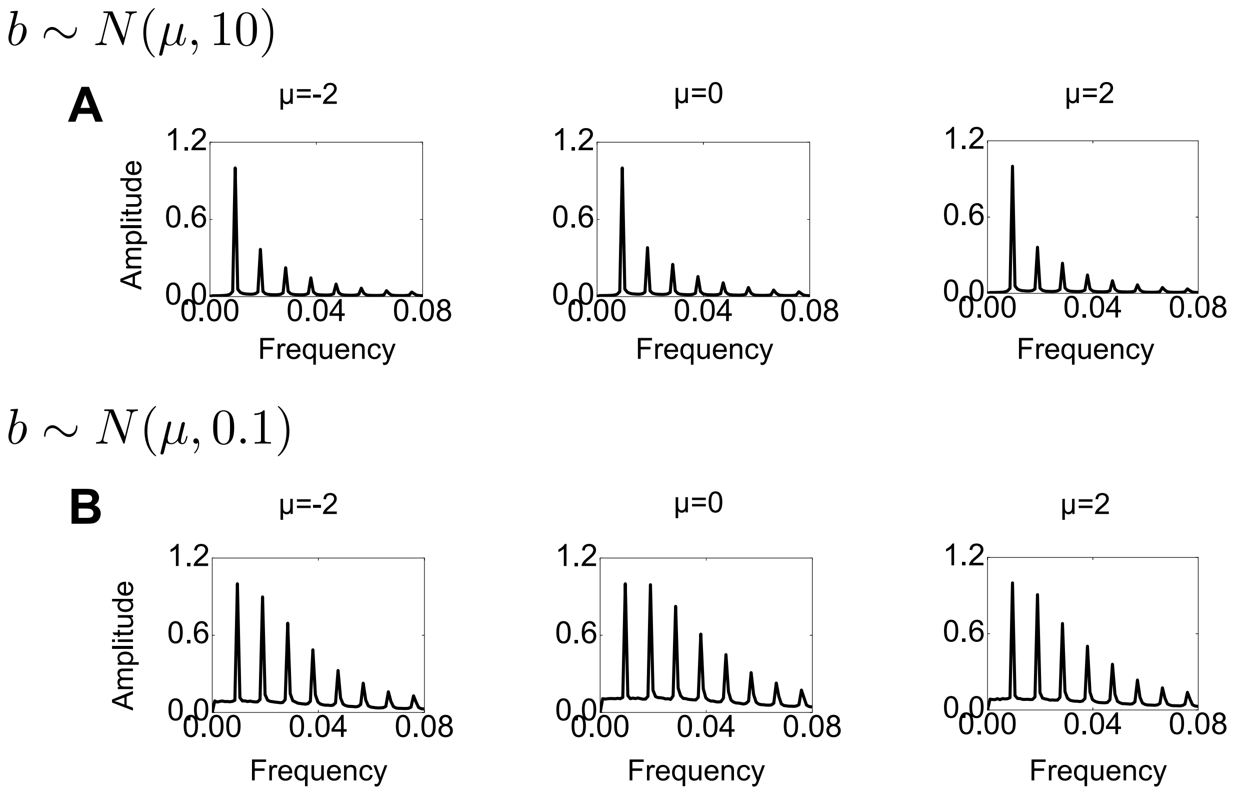

Figure S4. No significant effects of shifts of mean neuronal excitability on neural activity. (**A**) Results of the FT of context activity in the reproduction process of training data in each μ condition (heterogeneous condition $k = 10$). (**B**) Results of the FT of context activity in each μ condition (homogeneous condition $k = 0.1$). The results are averages of all 100 context neurons, 6 training datasets, and 8 trained networks. The amplitudes were mapped to values ranging from 0.0 to 1.0 by normalizing with the maximum value.

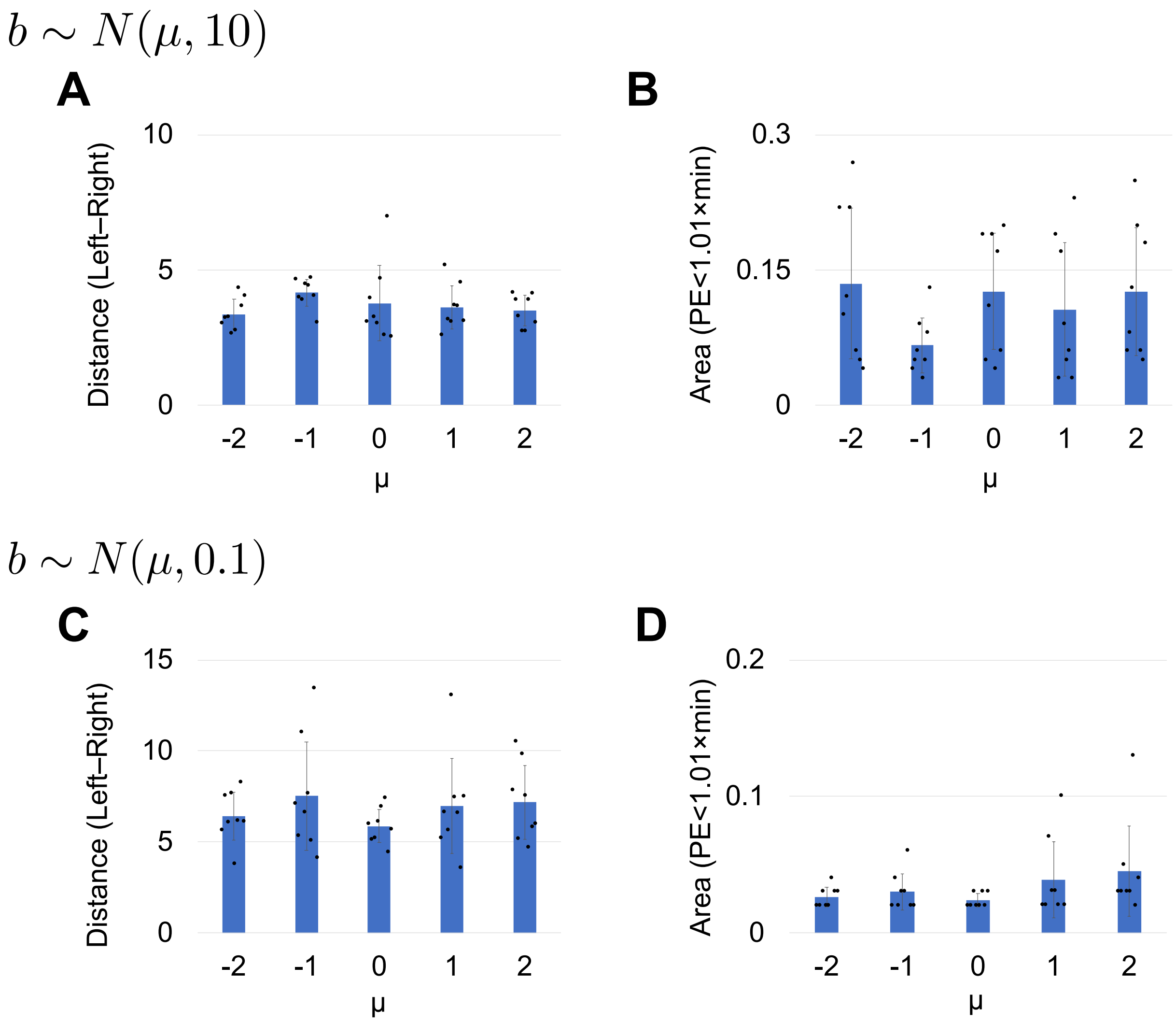

Figure S5. No significant effects of shifts of mean neuronal excitability on higher-level neural representation. (**A**) Distances between internal states of PB neurons corresponding to each behavior in each μ condition (heterogeneous condition $k = 10$). The distances were calculated using mean internal PB states acquired for 3 training datasets for each behavioral pattern. One-way ANOVA did not show a significant difference among the five network conditions ($F(4, 35) = 0.93$, $p = 0.46$). (**B**) Areas of PB activities at which prediction errors (PE) for training data were smaller than 1.01 times the minimum of the PE, varying with PB activities in each μ condition (heterogeneous condition $k = 10$). One-way ANOVA failed to indicate a significant difference among the five conditions ($F(4, 35) = 1.19, p = 0.33$). (**C**) Distances of PB activities coding learned behaviors in each μ condition (homogeneous condition $k = 0.1$). One-way ANOVA failed to indicate a significant difference among the five network conditions ($F(4, 35) = 0.67$, $p = 0.61$). (**D**) Areas of PB activities encoding learned behaviors in each μ condition (homogeneous condition $k = 0.1$). One-way ANOVA did not show a significant difference among the five network conditions ($F(4, 35) = 1.30$, $p = 0.29$). All results are averages from 8 trained networks and expressed as mean $\pm$ SD.

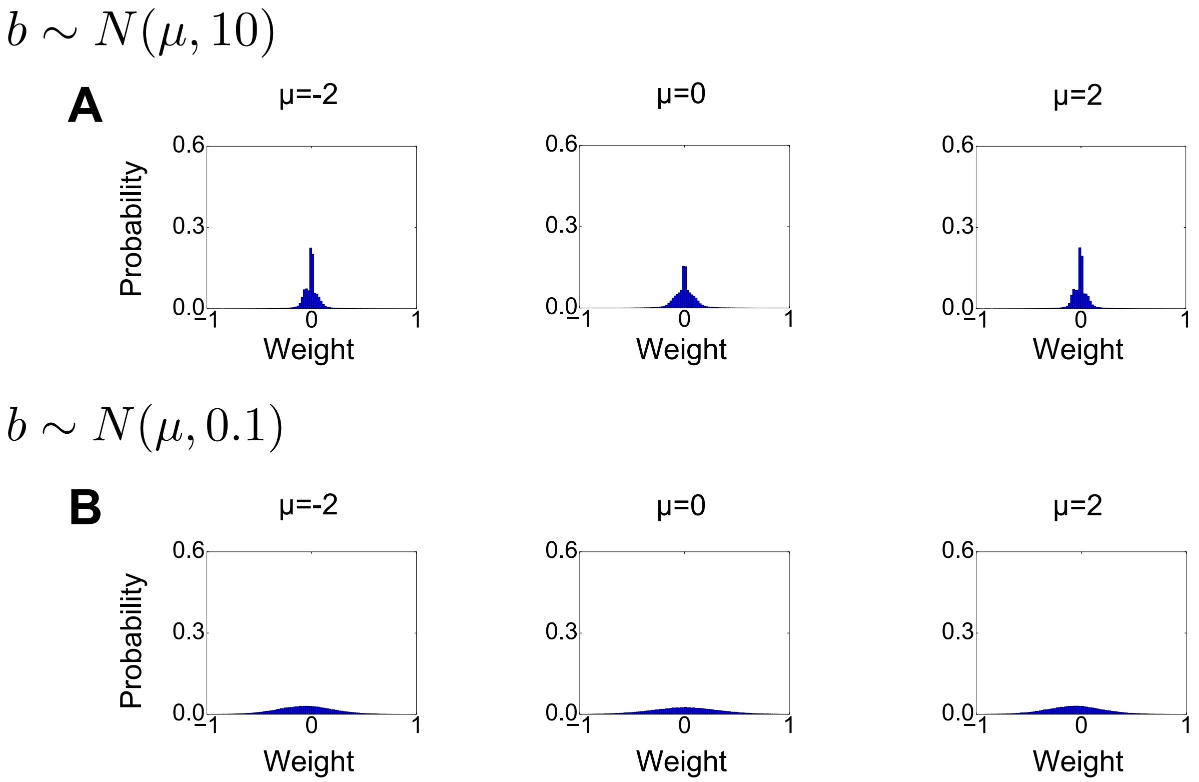

Figure S6. No significant effects of shifts of mean neuronal excitability on development of synaptic weights. (**A**) Distribution of synaptic weights after learning in each μ condition (heterogeneous condition $k = 10$). (**B**) Distribution of synaptic weights after learning in each μ condition (homogeneous condition $k = 0.1$). The results are averages from 8 trained networks.

2 SUPPLEMENTARY VIDEOS

Video S1. Successful real-time interaction by a robot controlled by a heterogeneous network. The robot could successfully pass the ball back to the experimenter. In addition, the robot could switch its action according to the environmental change. The generated time-series data through the task execution are shown in Figure 2B.

Video S2. Behavioral alterations in the real-time interaction observed in a robot controlled by a homogeneous network. The robot failed to return the ball to the experimenter, and the robot could not switch its action according to the environmental change. The generated time-series data through the task execution are shown in Figure 2C.

Video S3. Robot's action generated through mental simulation in a heterogeneous condition. The robot could stably reproduce the left behavior. Generated time-series data are shown in Figure 4B.

Video S4. Robot's action generated through mental simulation in a homogeneous condition. The robot could reproduce the left behavior, but the action seemed clumsy. The generated time-series data are shown in Figure 4C.
